# Mechanisms of the *Ping-wei-san plus* herbal decoction against Parkinson’s disease: Multiomics analyses

**DOI:** 10.3389/fnut.2022.945356

**Published:** 2023-01-04

**Authors:** Ding Li, Hong-juan You, Guo-jie Hu, Ru-yong Yao, An-mu Xie, Xiao-yuan Li

**Affiliations:** ^1^Department of Traditional Chinese Medicine, Affiliated Hospital of Qingdao University, Qingdao, Shandong, China; ^2^School of Basic Medicine, Qingdao University, Qingdao, Shandong, China; ^3^Medical Research Center, Affiliated Hospital of Qingdao University, Qingdao, Shandong, China; ^4^Department of Neurosurgery, Affiliated Hospital of Qingdao University, Qingdao, Shandong, China

**Keywords:** Parkinson’s disease, microbiome, metabolomics, tandem mass tag labeling, herbal, *Ping-wei-san plus*

## Abstract

**Introduction:**

Parkinson’s disease is a neurodegenerative disorder involving loss of dopaminergic neurons. Multiple studies implicate the microbiota-gut-brain axis in Parkinson’s disease pathophysiology. *Ping-wei-san plus* Herbal Decoction, a traditional Chinese medicine composition with beneficial effects in Parkinson’s disease, may have a complex array of actions. Here we sought to determine whether gut microbiota and metabolic pathways are involved in *Ping-wei-san plus* herbal therapy for Parkinson’s disease and to identify functional pathways to guide research.

**Methods and results:**

The model of Parkinson’s disease were induced with the rotenone. The *Ping-wei-san plus* group received the PWP herbal decoction for 90 days, after which all groups were analyzed experimentally. PWP herbal treatment improved motor behavior and emotional performance, balanced gut microbiota, and benefited dietary metabolism. Tandem Mass Tags mass spectrometry identified many differentially expressed proteins (DEPs) in the substantia nigra and duodenum in the PWP group, and these DEPs were enriched in pathways such as those involving cAMP signaling, glutamatergic synapses, dopaminergic synapses, and ribosome-rich functions in the gut. The PWP group showed increases in recombinant tissue inhibitors of metalloproteinase 3, and nucleotide-binding oligomerization domain, leucine rich repeat, and pyrin domain containing proteins 6 in the substantia nigra and decreased parkin, gasdermin D, recombinant tissue inhibitors of metalloproteinase 3, and nucleotide-binding oligomerization domain, leucine rich repeat and pyrin domain containing proteins 6 in the duodenum.

**Discussion:**

In conclusion, this study combined gut microbiota, metabolomics, and proteomics to evaluate the mechanism of action of *Ping-wei-san plus* on Parkinson’s disease and revealed that PWP herbal treatment modulated gut microbiota, altered metabolite biological pathways, and affected functional pathway protein expression in Parkinson’s disease mice, resulting in therapeutic effects.

## Introduction

Parkinson’s disease (PD) is a neurodegenerative disorder characterized by bradykinesia, resting tremors, muscle rigidity, and postural instability (1). The loss of dopaminergic neurons and the formation of alpha-synuclein (α-synuclein, AS)-containing Lewy bodies in the substantia nigra of the midbrain are considered to be the main pathophysiological characteristics of PD (2). Methods for the prevention of α-syn spread in the gut-to-brain axis are required to prevent neurodegeneration and behavioral deficits. More and more works have implicated the microbiota-gut-brain axis in Parkinson’s disease (3, 4). This axis is gaining traction in fields that investigate its biological and physiological basis. Communication along the gut-brain axis is a fundamental aspect of the synergy between microbiota and the host accessing gut-brain signaling pathways to modulate the host brain and behavior. The microbiota and the brain communicate with each other *via* various routes mediated by microbial metabolites such as short-chain fatty acids and branched-chain amino acids, including the immune system, tryptophan metabolism, the vagus nerve, and the enteric nervous system (5, 6). Recently, two live bacterial drugs [*Parabacteroides distasonis* MRx0005 and *Megasphaera massiliensis* MRx0029 (7)] for the treatment of PD received FDA approval for clinical use and marked the expansion of microbial therapy into the area of neurological disorders. Some doubt that intervention through the gut microbiome alone will be effective in treating PD, considering that, even if the disease does originate in the gut, microbial therapy is unlikely to restore dopamine neurons that have already been lost from the brain. To date, we believe that herbal medicine is a more promising complementary medicine than active bacterial replacement therapy because of its potential to regulate gut microbiota, reduce nervous system inflammation, protect neurons from oxidative stress damage, and restore intestinal barrier integrity (8).

Traditional Chinese medicine (TCM) is one of the most important components of complementary and alternative medicine (CAM). Traditional Chinese medicinal compounds (TCMCs) are a traditional combination of Chinese herbal medicines. According to historical records, multiple Chinese herbal medicines have been combined to exert more therapeutic effects by acting on multiple pathogenic mechanisms in the treatment of various diseases. In our clinical work, we prescribed medicines according to the symptoms of patients with PD and found that a classic TCMC prescription *Ping-wei-san* plus medicine (*PWP*, containing *Rhizoma Atractylodis* 12 g, *Magnolia officinalis var.* 9 g, *Pericarpium Citri Reticulata* 9 g, *Anemarrhena asphodeloides Bunge* 12 g, *Rhizoma Coptidis* 6 g, *Aucklandia lappa DC.* 9 g, *Cistanche deserticola Y.C.Ma* 15 g, *Radix Glycyrrhizae* 3 g, components are classified)^1^ ^2^ can not only methods for the prevention of α-syn spread in the gut-to-brain axis are required to prevent neurodegeneration and behavioral deficits. More and more works have implicated the microbiota-gut-brain-axis in Parkinson’s disease effectively improve the symptoms of patients with PD, but also improve the prognosis of patients with PD. According to previous studies, TCMCs play a therapeutic role in the treatment of neurological diseases such as PD and depression through the hypothalamic-pituitary-adrenal (HPA) axis or antioxidative, ferroptosis and autophagy, etc. (9–12). Because of the broadly effective active components of TCMCs, their mechanism of action is extremely complex, which is undoubtedly also the advantage of TCMCs in treating diseases. To better understand the complex effects of PWP, we used proteomic (“tandem mass tags” or TMT mass spectroscopy, microbial community analysis (16s RNA sequencing), and metabolomic techniques to gain mechanistic insight into the pathogenesis of PD and how TCMCs exert their therapeutic effects.

Therefore, our study focused on understanding the mechanisms by which herbs regulate the microbiota-gut-brain axis and sought to elucidate the effectiveness of microbial-based intervention and therapeutic strategies from multiple perspectives.

## Materials and methods

### Materials

Female C57BL/6 mice (12–14 weeks, 22–26 g) were purchased from Beijing Vital River Laboratory Animal Technology Co., Ltd., for use in experiments. Herbal medicated feed and AIN-93 standard feed were purchased from Nantong Trofi Feed Technology Co., Ltd., China. Most reagents used in this study were purchased from MedChemExpress. cn and Abcam.cn. Herbal medicine were purchased from Shanghai Pharmaceutical Co., Ltd.

### Experimental design

**FIGURE 1:**
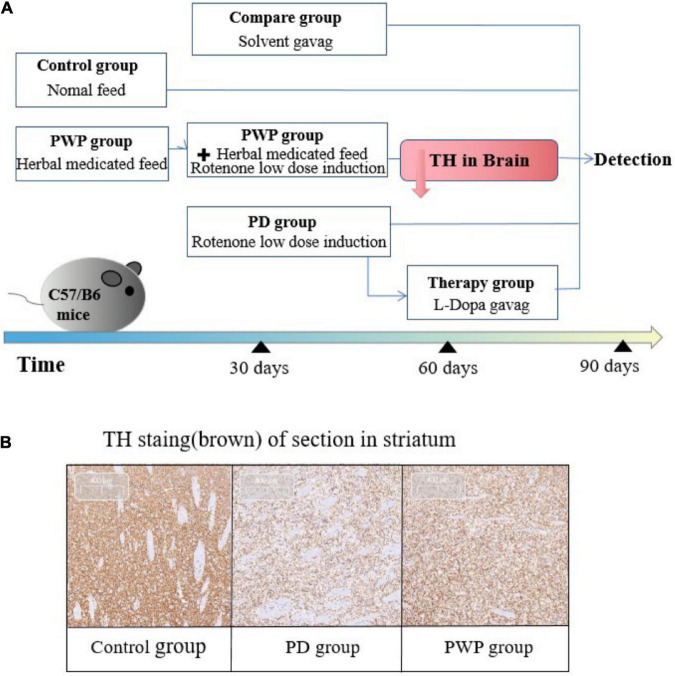
Experimental process. **(A)** Mouse experiment study design. **(B)** Confirmation of PD animal model establishment: TH immunohistochemical staining in striatum on the 60th day.

The PWP herbal decoction was prepared by the Pharmacy Department of the Affiliated Hospital of Qingdao University. All mice were raised in an environment with a 12-h light/dark cycle at a temperature of 20 ± 1°C with available food and water. All experiments were conducted according to the principles established for the care and use of laboratory animals by the National Institutes of Health and approved by the Medical Professional Committee of the Affiliated Hospital of Qingdao University (QYFY WZLL 26506). All mice were randomly divided into the following groups (*n* = 10–12 per group): control, PD model, PWP, L-Dopa therapy and solvent comparison groups. Mice in the control group were fed American lnstitute of Nutrition-93 standard feed daily at a dose of 6-7 gram per mouse. Mice in the PWP group were given herbal medicated feed at a dose of 6-7 gram per contained the herbal dosage of 975 mg/kg every day for 30 consecutive days. Then, mice in the PD group and PWP group were given rotenone solvent gavage at a dose of 0.75 mg 7.2 mg, 10% DMSO 360 μl, PEG300 1,440 μl, Tween-80 180 μl/kg, once a day in the morning. Rotenone Solvent, containing rotenome 7.2 mg, 10% DMSO 360 μl, PEG300 1,440 μl, Tween-80 180 μl, normal saline 1,620 μl (MedChemExpress, China). Mice in the solvent comparison group were gavaged a solvent solution in the morning once daily. The groups were processed for two months. After treatment, we performed substantia nigra tyrosine hydroxylase (TH) immunohistochemical staining and behavioral tests to detect whether the PD model was successful. Next we removed 4 mice from the model group as the L-Dopa treatment group and performed L-Dopa (MedChemExpress, China) gavage in the afternoon once daily at a dose of 20 mg/kg, dissolved in PBS at a concentration of 1 mg/mL and dissolved by ultrasonic. Intestinal permeability tests were performed before death. Fecal samples were collected from all mice and used for 16S rRNA gene sequencing and metabolomic analysis. After behavioral testing, the mice were deeply anesthetized with isoflurane inhalation followed by 10% chloral hydrate and euthanized by cervical dislocation. The substantia nigra and duodenum were isolated for TMT, enzyme-linked immunosorbent assay (ELISA), and western blotting (WB) (Figure 1A).

### Immunohistochemistry

Mice were transcardially perfused with 0.9% saline, followed by 4% paraformaldehyde for tissue fixation. The corpus striatum was dissected from brain according to the mouse brain atlas, and immediately frozen in liquid nitrogen until further use. Striatal tissues were fixed in cold 4% paraformaldehyde and embedded in paraffin. For IHC, the brain tissue slide was deparaffinized. Sections were incubated with primary anti-body: anti-Tyrosine Hydroxylase (1:500; Abcam) for 60 min followed by goat-anti mouse IgG (1:1,000; Abcam) for 30 min at room temperature and visualized with diaminobezidin (DAB) reagent. After counterstaining with hematoxylin, all slices were detected using an optical microscope(CX31;Olympus, Tokyo, Japan).

### Behavioral testing

#### Open field test

Mice were acclimated 7 days prior to the experiment to familiarize them with the investigator, and then weighed and placed in the testing room before testing. The test mouse was placed onto the central zone of the open field box and started on the timer to allow the mouse to explore the test area for five min. The entire area of the box was cleaned before proceeding to the next test mouse.

#### Rota-rod test

After completing the open field test, the Rota-Rod test was performed the following day. The Rota-Rod speed was set to accelerate from 5 to 50 rpm over 150 s, and the time and speed (in rpm) required for the animal to fall from the Rota-Rod were measured. Before starting the Rota-Rod study, all mice were allowed to walk for 1 min at the same rod speed (5 rpm). After 1 min, the Rota-Rod speed was gradually increased to 50 rpm for 2 min, and the rod speed was maintained until the mice fell off the bar. The trials were performed three times and were conducted every 20 min.

### 16S rRNA microbial community analysis

To analyze the taxonomic composition of the microbial community, 16S rRNA sequencing analysis was performed. Briefly, PCR amplification was performed, the purified amplicons were pooled, and paired-end sequencing was performed. The raw data were then analyzed. Sequencing of the 16S rRNA amplicon (V3-V4 region) was performed using MiSeq 300PE (Illumina MiSeq System), resulting in a total of 1715085 sequence reads (723701476 bases) and an average sequence length of 421 bp. Bioinformatics was performed using Mothur and QIIME2.0 software and included taxonomic annotation, taxonomy-based comparisons at the genus level, β-diversity analysis (principal coordinates analysis), and dissimilarity analyses. Detailed sequencing analysis procedures are available in the Supplementary materials and Methods.

### Fecal metabolomic analysis

Liquid chromatography mass spectrometry (LC–MS) analysis was performed by Majorbio Bio-Pharm Technology (Shanghai, China). The fecal samples of the control, PD, and PWP groups were subjected to liquid chromatography for component separation; a single component entered the ion source of the high vacuum mass spectrometer for ionization and was separated according to the mass-to-charge ratio (m/z) to obtain a mass spectrum. Finally, the mass spectra of the samples were analyzed. The qualitative and quantitative results of the samples were obtained.

The LC–MS data from fecal pellets were processed using the Majorbio Cloud Platform (Shanghai, China), and metabolites were identified. Normalized data were visualized by orthogonal partial least squares-discriminant analysis (OPLS-DA) using the ropls package in R. The ellipses in the OPLS-DA plots were employed to characterize metabolic perturbations among groups in a Hotelling T2 region with a 95% confidence interval threshold.

The variable importance in projection (VIP) was calculated based on the OPLS-DA model to identify significant metabolites with a VIP > 1.0 and *P*-value <0.05. The KEGG^3^ database was used to explore related metabolic pathways.

### Tandem mass tags labeling, high performance liquid chromatography fractionation and LC-MS/MS analysis

After trypsin digestion of the protein samples, the peptide was desalted using a Strata X C18 SPE column (Phenomenex) and vacuum-dried. The peptide was reconstituted in 0.5 M TEAB and processed for the 6-plex TMT kit according to the manufacturer’s instructions. Briefly, the labeling reagent was thawed, dissolved in acetonitrile, mixed with the peptides, and incubated at room temperature for 2 h. The labeled peptides were mixed, desalted, and lyophilized under vacuum.

The labeled peptides were fractionated into 60 fractions by high pH reverse-phase HPLC using an Agilent 300Extend C-18 column (5 μm particle size, 4.6 mm ID, 250 mm length) with a gradient of 8% to 32% acetonitrile (pH 9.0) over 60 min. Then, the 60 fractions were combined into 18 fractions, and each fraction (volume of 800 μL) was dried by vacuum centrifugation pending MS analysis. LC-MS/MS analysis of labeled peptides was performed as previously described (13). The related technical support and bioinformatics analyses were provided by Jingjie PTM BioLabs (Hangzhou, China).

### Western blot

The protein expression of gasdermin D and parkin in the substantia nigra and duodenum was detected by western blotting. Total protein was extracted from the substantia nigra and duodenum tissues in RIPA lysis buffer containing protease inhibitors. Protein concentrations were determined by a BCA assay kit (Beyotime Institute of Biotechnology, Shanghai, China). Protein samples were mixed with loading buffer (Beyotime, Shanghai), and then the mixtures were boiled at 98°C for 5 min. Equal amount of protein (40 μg/lane) was loaded on an 4-10% of sodium dodecyl sulfate-polyacrylamide gel electrophoresis at a constant voltage of 90 V. After 180 min, the gels were transferred to a poly (vinylidene difluoride) membrane (Millipore Corp., MA) and blocked with 5% non-fatty milk or BSA in Tris-buffered saline Tween-20 (TBST) solution at room temperature for 1 h. The membranes were then incubated with the and incubated with primary antibodies respectively anti-GSDMD (1:2,000; Abcam), anti-Parkin (1:1,500; Abcam), anti-β-actin (1:1,500; Abcam) at 4°C overnight, washed three times with TBST. The membranes were further incubated with 1:5,000 dilution of the horseradish peroxidase-conjugated secondary antibody for 1 h and then washed three times with TBST again. Specific protein bands were then detected using ECL reagent (Millipore Corp., MA) and quantified using Tanon 5,200 Chemiluminescence Imaging System (Shanghai, China),quantified by Image J software (Rawak Software Inc., Germany).

### ELISA

The protein expression of recombinant tissue inhibitors of metalloproteinase 3 (TIMP3), caspase-1(Casp-1), inter alpha-globulin inhibitor H3 (ITIH3), and nucleotide-binding oligomerization domain, leucine rich repeat and pyrin domain containing proteins-6 (NLRP6) in the substantia nigra and duodenum was detected using ELISA kits. Briefly, 100 μl of horseradish peroxidase (HRP)-labeled detection antibody was added to each well of standard and sample 96-well plates, and they were then sealed with a sealing film and incubated at 37°C for 60 min. After five washes, 50 μl of substrate A was added to each well, followed by 50 μl of substrate B, shaken gently, mixed, and incubated at 37°C for 15 min in the dark. 50 μl of stop solution was added to each well to terminate the reaction. The optical density (OD) of each well was measured using a microplate reader at a wavelength of 450 nm within 15 min of reaction termination.

### Intestinal permeability evaluation

Mice fasted for four hours were gavaged with 0.5 ml of fluorescein isothiocyanate conjugated-dextran (FITC-dextran; FD-4, 22 mg/ml, molecular mass 4.4 kD, Sigma-Aldrich). Blood samples were collected after 3.5 hours and diluted 1/100 in PBS to measure fluorescence intensity using a fluorospectrophotometer with an excitation wavelength of 485 nm and an emission wavelength of 528 nm. FITC-dextran concentrations were determined from a standard curve generated using serial dilutions of FITC-dextran.

### Statistical analysis

Statistical analysis was conducted using SPSS for Windows (version 19.0; SPSS Inc., Chicago, IL, USA). All data are presented as the mean ± SD. The significance of the differences between the two groups was analyzed using Student’s unpaired *t*-test, and multiple comparisons were analyzed using one-way ANOVA followed by Dunnett’s *post hoc* test. The differential abundances of genera and metabolites were determined using non-parametric tests, including the Wilcoxon rank-sum and Kruskal-Wallis H tests. Correlations among fecal metabolites, 16S levels, and physiological and biochemical indices were evaluated using both the Pearson correlation coefficient and Spearman rank correlation. *P*-values <0.05 was considered statistically significant.

## Results

### Establishment of the PD disease model and behavioral responses related to motor and dopaminergic activities

**FIGURE 2:**
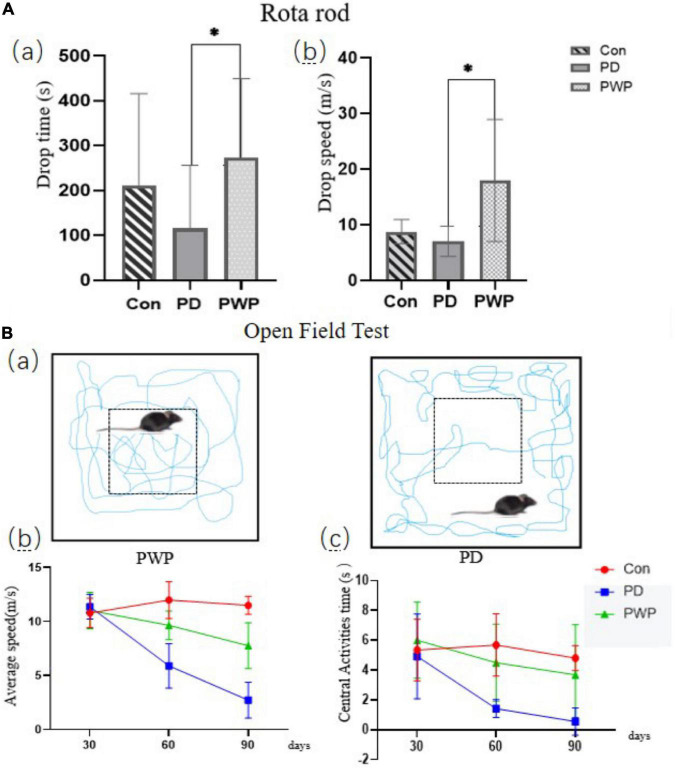
Behavioral testing. **(A)** Rota-Rod test performed on the 90th day, including drop time (s) and drop speed (m/s). **(B)** Open field testing performed on the 30th, 60th, and 90th days; motion track simplified diagram showing PD mice (right) and PWP mice (left) with movement (blue trails) in the peripheral or central regions; test data included average speed and central activities time. *Means that *p* < 0.1 for significant differences.

The IHC results showed that the TH content in the striatum of the PD group decreased significantly after 30 days of low-dose rotenone induction, which confirmed the establishment of the PD disease model (Figures 1A,B). By the way PWP attenuated these effects. The pole test results showed that the PD mouse model exhibited exercise fatigue and bradykinesia, whereas PWP-treated mice partially recovered their motor skills. The mice treated with PWP recovered from movement disorders within 90 days, close to the control group condition (Figure 2A). Behavioral recovery does occur in this animal model with PWP intervention. Mice in the control and PWP groups spend more time in the central, open area of the box. Mice in the PD group that were stressed showed less activity in the open field, increased stereotypical behavior, preferred staying close to the walls, and traveled more in the periphery (Figure 2B).

### *Ping-wei-san plus* herbal medicines alter the fecal microbiota of Parkinson’s disease mice

**FIGURE 3:**
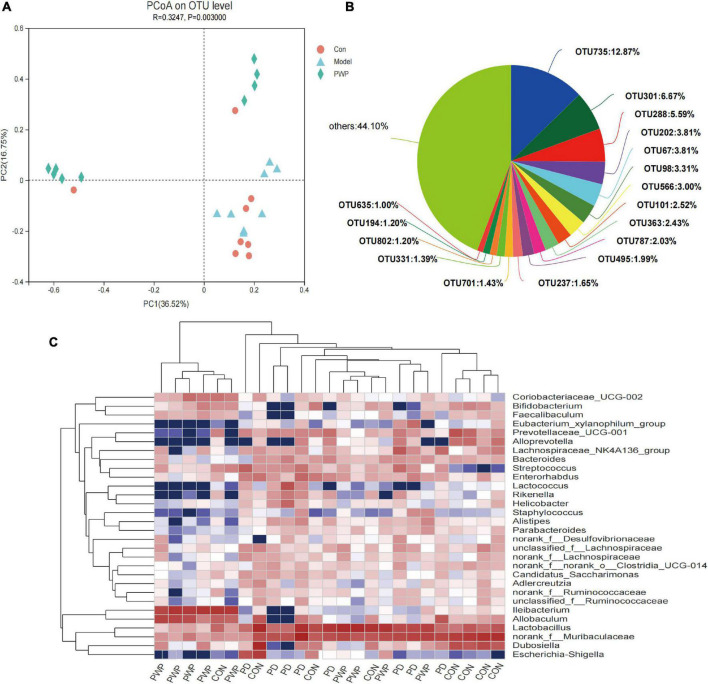
The result of 16S microbiome analysis. **(A)** PCoA on OTU level on identified β-microbial diversity in the control, PD, and PWP groups. **(B)** The microbial diversity on OTU level of the control, PD, and PWP groups. **(C)** Community heatmap analysis by Genus.

**FIGURE 4:**
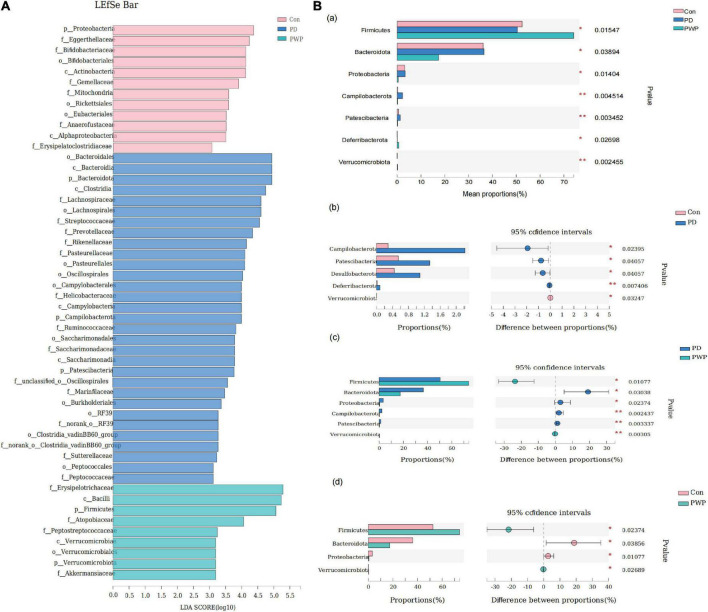
The result of 16S microbiome analysis. **(A)** LEfSe bar of control, PD, and PWP groups. **(B)** Results of Kruskal-Wallis H test, showing difference between groups.

16S rRNA analysis revealed 317 distinct taxa, and we further analyzed 243 taxa. Principal coordinate analysis (PCoA) was used to identify the significance of the differences and highlight within-replicate variations (Figures 3A–C). LEfSe analysis (LDA threshold of 2) revealed that *Actinobacteria* and *Verrucomicrobiota* were significantly enriched in the feces of PWP-fed mice compared to the PD group. Conversely, *Bacteroidota* and *Campilobacterota* were enriched in the PD group (Figure 4A). Species difference analysis revealed significant differences in the microbial taxa at the family level among the three groups. The results showed that the abundance of *Firmicutes* and *Verrucomicrobiota* in the PWP-fed group was significantly higher than that in the PD group (*P < 0.05, ^**^P < 0.01, Wilcoxon rank-sum test), while *Bacteroidota*, *Proteobacteria*, *Campilobacterota*, and *Patescibacteria* in the PWP-fed group were significantly lower than those in the PD group (*P < 0.05, ^**^P < 0.01, Wilcoxon rank-sum test). The abundances of *Deferribacterota*, *Campilobacterota*, *Patescibacteria*, and *Desulfobacterota* in the PD group were significantly higher than those in the control group (^**^P < 0.01, *P < 0.05, Wilcoxon rank-sum test) (Figure 4B).

### *Ping-wei-san plus* herbal medicines alter the fecal metabolomic of Parkinson’s disease mice

**FIGURE 5:**
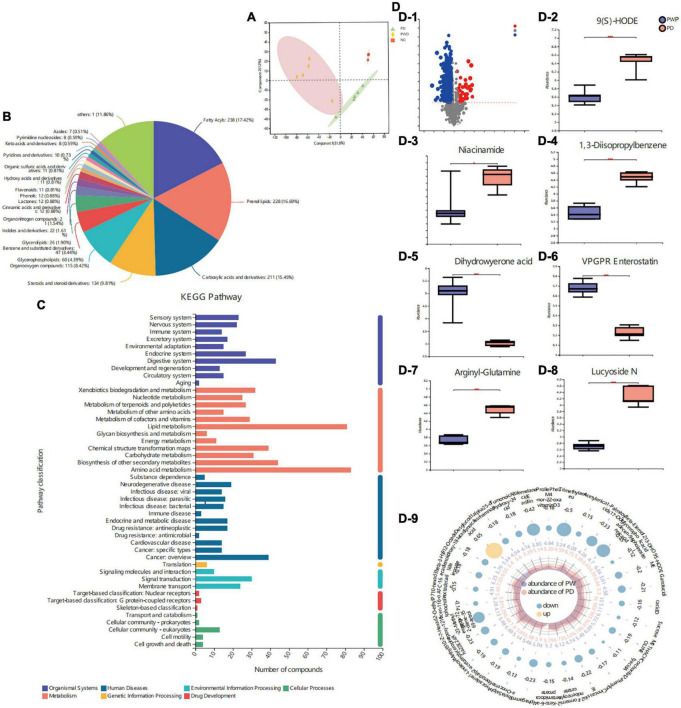
The result of Fecal Metabolomics. **(A)** Principal component analyses (PCAs) on identified metabolites in control, PD, and PWP groups. **(B)** Pie chart based on counts of HMDB chemical taxonomy (class) for all metabolites detected in this study class. **(C)** KEGG pathway classification of metabolites detected and annotated. **(D)** Discrepant metabolites in comparison of the PD group and PWP group.

The metabolite profiles of fecal samples were analyzed using LC-MS. The metabolites in the control, PD, and PWP groups were well separated, with fractions 1 and 2 explaining 51.8 and 12% of the variance, respectively (Figure 5A). Based on the HMDB and KEGG database, the 97 molecules compounds were identified including fatty acyls, prenol lipids, flavonoids, carboxylic acids and derivatives (Figure 5B). Among the seven categories in KEGG metabolic pathway, most abundant metabolites were annotated in amino acid metabolism, lipid, biosynthesis of other secondary metabolites, digestive system, cancer: overview, chemical structure transformation maps, xenobiotics biodegradation and metabolism, carbohydrate metabolism, signal transduction (Figure 5C). The content of Lucyoside N, Arginyl-Glutamine, Niacinamide, 1,3-Disopropylbenzene, 9(S)-HODE of fecal metabolites were higher in the PD group than that in the PWP group, and the content of VPGPR Enterostatin and dihydrowyerone acid of fecal metabolites were higher in the PWP group than that in the PD group (*P* < 0.001,Figure 5D).

**FIGURE 6:**
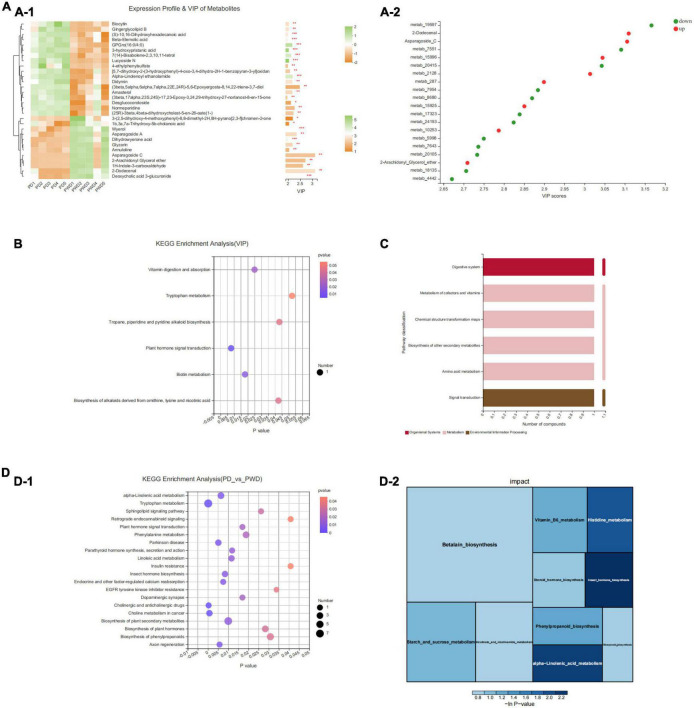
The result of Fecal Metabolomics. **(A)** A heatmap of differentially expressed metabolites between the PD and PWP groups showing the top 30 significantly differentially expressed metabolites. **(B)** Bubble map showing the results of KEGG pathway enrichment analysis. **(C)** Secondary metabolic classification of KEGG functional pathway. **(D)** KEGG enrichment analysis of the PD and PWP groups.

We identified the top 30 differential metabolites in the PD group versus the PWP group, as well as the PD group versus the control group by VIP based on the OPLS-DA model (Figure 6A). The results of KEGG pathway enrichment showed that PWP mainly plays a role in the treatment of PD through the biosynthesis of alkaloids, such as ornithine, lysine, and nicotine. The metabolic pathways involved include acid, biotin metabolism, phytohormone signaling, tropane, piperidine, pyridine alkaloid biosynthesis, tryptophan metabolism, and vitamin digestion and absorption (Figure 6B). The results of the KEGG functional pathway analysis showed that PWP treatment of PD mainly exerted its therapeutic effect through three metabolic pathways: organic system, metabolism, and environmental information processing (Figure 6C). Concomitantly, we found the top five significant difference of KEGG pathway enrichment were cholinergic and anticholinergic drugs, plant hormone signal transduction, dopaminergic synapse, axon regeneration and endocrine and other factor-regulated calcium reabsorption (*P* = 0.12,Figure 6D).

### Combined analysis of fecal metabolomic with gut microbes

**FIGURE 7:**
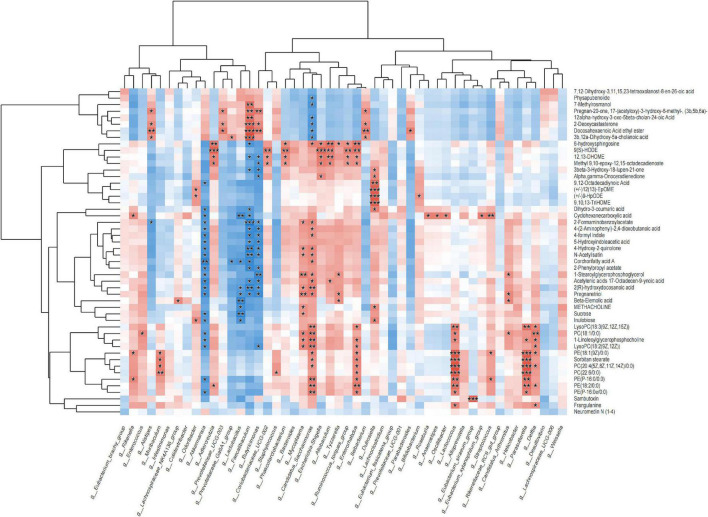
Correlation between metabolics and 16S microbiome analysis.

Fecal metabolomics combined with 16S analysis showed that the content of Bacteroides in the PD group was higher than that in the PWP group, and the VIP differential metabolite 9-hydroxy-10E, 12Z-octadecadienoic acid (9-HODE, 9(S)-HODE) increased the content of Bacteroides (Figure 7). The 9 (S)-HODE content in the feces of the PD group was higher than that in the PWP group (Figure 5D-2).

### Brain and gut proteomic bioinformatics

**FIGURE 8:**
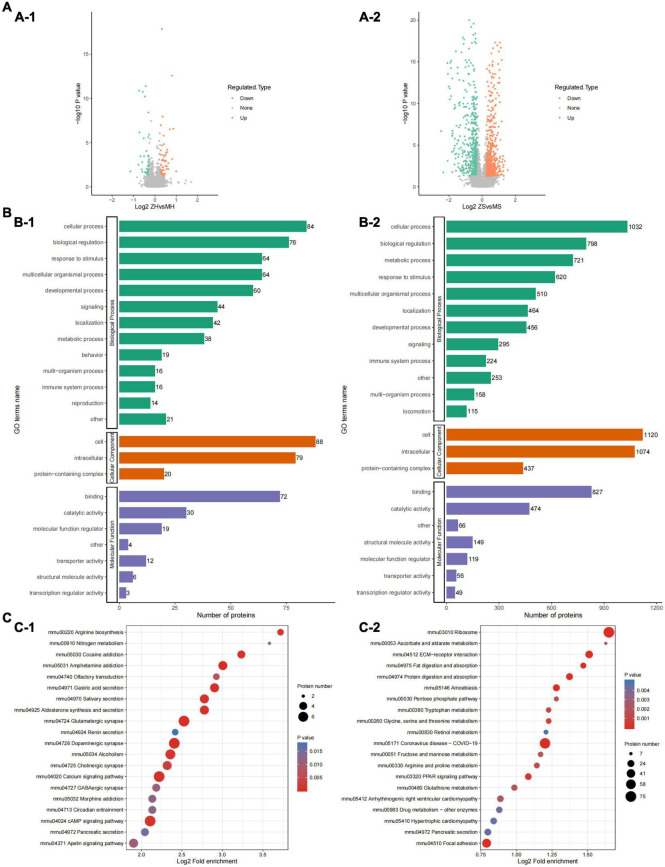
The results of TMT labeling studies. **(A)** Volcano plot of differential proteins between the PD and PWP groups. **(A-1)** Differential proteins in substantia nigra tissue. **(A-2)** Differential proteins in duodenal tissue. **(B)** Bar plot of GO terms of the PD and PWP groups. **(B-1)** The result were enriched in substantia nigra tissue. **(B-2)** The result were enriched in duodenal tissue. **(C)** Bar plot showing the result of KEGG pathways between the PD and PWP groups. **(C-1)** KEGG pathways in substantia nigra tissue. **(C-2)** KEGG pathways in duodenal tissues.

**FIGURE 9:**
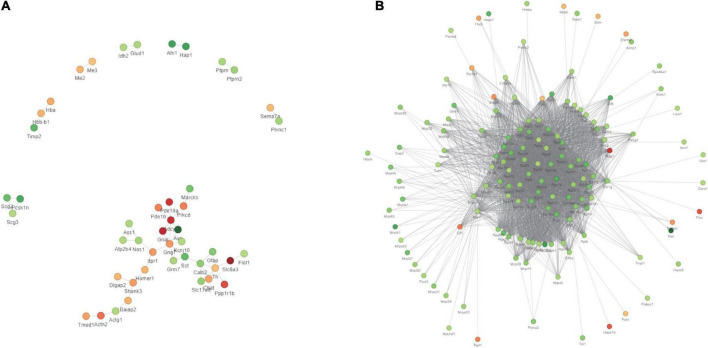
The results of TMT labeling studies. The PPI network of DEPs between the PD and PWP groups. **(A)** DEPs in substantia nigra tissues. **(B)** DEPs in duodenal tissues.

We first obtained data regarding differentially expressed proteins (DEPs) involved in PWP-treated PD in the substantia nigra and duodenal tissues by differential analysis. In the substantia nigra tissue, we identified 98 upregulated and 98 downregulated DEPs (Figure 8A-1). In duodenal tissue, we found 624 upregulated DEPs and 591 downregulated DEPs (Figure 8A-2). We then analyzed the biological effects and signaling pathways involved in PWP treatment of PD using Gene Ontology (GO) enrichment analysis and KEGG enrichment analysis. In biological processes (BP), DEPs in the substantia nigra tissue and duodenal tissue are enriched in multiple identical BP, such as cellular processes, biological regulation, response to stimulus, and localization. In the cellular component (CC), DEPs in the substantia nigra and duodenal tissues were enriched in three identical CC, including cell, intracellular, and protein-containing complexes. In molecular function (MF), DEPs in the substantia nigra tissue and duodenal tissue were enriched in multiple identical MF, such as binding, catalytic activity, molecular function regulator, and structural molecule activity (Figure 8B). In the KEGG pathway database, the DEPs in the substantia nigra tissue were mainly enriched in the calcium signaling, cAMP signaling, glutamatergic synapse, dopaminergic synapse, and alcoholism pathways (Figure 8C-1). The DEPs in duodenal tissue were mainly enriched in the ribosome, coronavirus disease (COVID-19), and focal adhesion pathways (Figure 8C-2). Finally, we constructed protein-protein interaction (PPI) networks of DEPs in different tissues using the STRING database, with a confidence score >0.7 as the filter condition. We demonstrated PPI network of DEPs in the substantia nigra tissue and in the duodenal tissue (Figure 9).

### Protein expression in brain and gut by western blotting and ELISA

**FIGURE 10:**
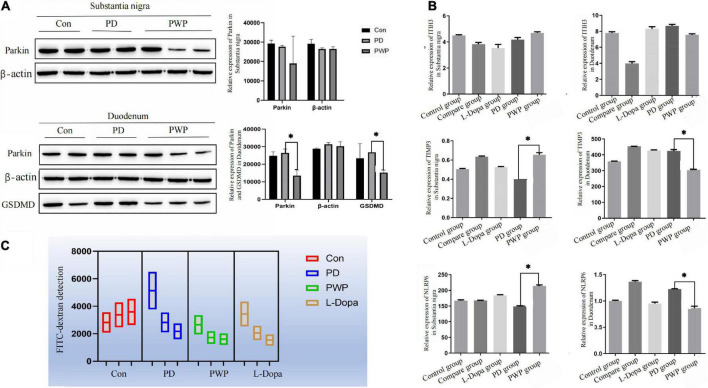
The result of protein expression in Brain and gut by WB and ELISA, as well as FITC-dextran concentrations test. **(A)** WB showing the expression of Parkin, and GSDMD in the substantia nigra and duodenum. **(B)** ELISA results showing the expression of TIMP3, NLRP6, and ITIH3 in the substantia nigra and duodenum. **(C)** FITC-dextran concentrations test in the control, PD, L-Dopa therapy, compare and PWP groups. *Means that *p* < 0.1 for significant differences.

After identifying the differentially expressed proteins in the substantia nigra and duodenum by TMT, we performed WB and ELISA detection of some of these proteins to evaluate the pathway by which PWP promotes PD motility and gastrointestinal function through gut microbes. We found that in the substantia nigra, PWP increased the expression of TIMP3, and NLRP6, while in the duodenum, PWP decreased the levels of Parkin, GSDMD, TIMP3, and NLRP6 (Figures 10A,B).

### Intestinal permeability test

Differences in the expression of inflammatory factors were observed between the groups in the TMT study. To verify whether inflammatory injury caused changes in intestinal permeability in PD mice, the FITC-dextran flux assay was used to measure permeability and evaluate intestinal epithelial function. PWP herbal stimulation resulted in an increase in intestinal permeability, which was reflected by an increase in the transmembrane flux of FITC-dextran (Figure 10C).

## Discussion

Due to the multi-component and multi-target properties of TCMCs, TCMCs are also recognized as one of the most important treatments in the clinical setting, not only playing the main therapeutic role for some diseases such as frailty, but also assisting in the treatment for others diseases such as COVID-19 (12). An US study claimed that Chinese herbal medicine was making its way into mainstream Western medicine. Patients may discover *Ping-Wei-San* in their search for a substitute for cisapride, which was partially withdrawn from the US market in July 2000 (14). One study identified patterns of TCM use including *Ping-Wei-San* (usage of 2.4%) in colon cancer patients post-surgery in Taiwan. The herbal ingredients were most commonly used for stimulate ghrelin secretion to increase food intake and had potential anti-tumor effect (15). Findings from A Matched cohort study demonstrated that adding Chinese herbal medicines, e.g., *Ping wei san* to conventional treatment significantly reduces depression risk among patients with insomnia (16). However, the mechanism of action of TCMCs is difficult to explain precisely because of its multicomponent and multitarget properties. TCM regulates multiple systems in the human body, such as the gut microbiota, proteomics, and metabolomics, to play a therapeutic role (17–19). Therefore, this study combined gut microbiota, proteomics, and fecal metabolomics to comprehensively explore the potential mechanism of PWP in the treatment of PD from multiple perspectives and to evaluate evidence that PWP can effectively treat PD. In this study, we verified and explained the mechanism by which PWP treats and alleviates PD from multiple aspects.

First, we demonstrated that the gut bacterial community composition was different between the control, PD model, and PWP groups, belonging to multiple operational taxonomic unit (OTU) types, of which OTU735 accounted for the largest proportion. The top seven differences in gut flora between the three groups were associated with PD and the brain-gut axis (4), including *Firmicutes*, *Bacteroidetes*, *Campylobacter*, *Patescibacteriota*, *Desulfobacteriota*, *Verrucomicrobiota*, and *Deferribacterota*, which has been proved that the treatment of PWP from gut flora partly in previous studies (20–23). *Bacteroidota*, *Verrucomicrobiota*, and *Campylobacter* have been found to be an important part of the brain-gut-microbiota axis in PD. *Patescibacteriota* have been shown to play a role in the brain-gut-microbiota axis to promote the progression of brain and emotion-related diseases such as depression and brain glioma (24, 25). In addition, *Campylobacter*, *Patescibacteriota*, and *Verrucomicrobiota* were also the top three different gut flora between the PD and PWP groups, which may be the main mediators of the PWP herbal effect involving restore mitochondrial function and is neuroprotective.

Second, fecal metabolomics was used to explore the treatment mechanism of PWP acting on PD, and we found that PWP has an effect on multiple metabolites in the treatment of PD. The correlation results showed that there was a clear correlation between gut flora and fecal metabolomics. Among the differentially expressed fecal metabolites, alpha-linolenic ethanolamine has been reported to be associated with PD, a metabolite with neuroprotective effects in PD and stroke (26). 9(S)-HODE is one of the oxidation products of linoleic acid. In this experiment, under the action of PWP, both the content of 9(S)-HODE and bacteroides decreased. The former suggested that lipid peroxidation levels was low *in vivo*. Previous studies have found bacteroides trigger lipid peroxidation (27). Even though, it has been a long way to fully explore the interaction relationships between PWP, gut flora, and fecal metabolomics. In general, the holistic concept of TCM means that TCM not only treats PD from the nervous system but also exerts potential regulatory effects by regulating other systems (28); pathway classification and KEGG enrichment analysis also proved this.

Next, we used proteomics to elucidate the mechanism of action of PWP in the substantia nigra and duodenum of PD mice. As a mitochondrial electron transport chain complex I inhibitor, rotenone induces apoptosis by enhancing the production of mitochondrial reactive oxygen species. Intake of PWP herbs reduced the rotenone-induced loss of dopaminergic neurons in PD mice, as evidenced by TH immunostaining in the striatum. Although affected by the blood-brain barrier, some active ingredients still act on the substantia nigra-related proteins (29). The results of the enrichment analysis indicated the existence of multiple identical biological processes and signaling pathways, which also proved the effect of PWP on PD through the brain-gut axis. PPI also showed that centriole proteins, such as PDE10A, GNAL, SLC6A3, PPP1R1B, and ADCY5, are regulated by PWP in the substantia nigra. According to the research reports of central proteins, it is proven that PWP exerts therapeutic and regulatory effects through mechanisms such as regulating dopamine, dystonia, microglial activation and autophagy (30). The effects of rotenone in PD mice mainly involve mitogen-activated protein kinase (MAPK), which increases the levels of excitatory amino acid neurotransmitters (31, 32). Nod-like receptor 6 (NLRP6) is located at the intestinal epithelial barrier and is involved in sensing and maintaining the naturally colonized bacteria of the gut microbiome. NLRP6 plays a key role in regulating inflammation and host defense against gut microbes. Different studies have found that deubiquitination of NLRP6 prevents overproduction of interleukin 18 (IL-18) in the colonic mucosa; and when induced, NLRP6 undergoes phase separation to activate the inflammasome immune response (33). We found that NLRP6 expression was reduced in the duodenum of PWP intake mice. Gasdermin D (GSDMD) is a direct executor of inflammatory caspase-induced pyroptosis by driving mucin secretion through calcium-dependent sciderin-mediated cortical F-actin breakdown (34). TMT found that intestinal gasdermin D (GSDMD) was increased in PD mice compared with that in normal mice, and WB results showed that intestinal GSDMD expression decreased after 90 days of PWP intake, which might reveal PWP playing a role in shaping intestinal mucosal homeostasis.

## Conclusion

This study combined gut microbiota, metabolomics, and proteomics to explore the mechanism of action of PWP on PD mice. The results reveal that PWP herbal treatment modulates gut microbiota abundance, alters metabolite biological pathways, and affects functional pathway protein expression in PD mice, resulting in therapeutic effects. Although we are at the beginning of understanding the effect of PWP herbs in the microbiome that corresponds to functionally, the findings still provide new leads and testable hypotheses on the treatment of PD. This study paves the way for novel therapeutic strategies for the treatment of PD using Chinese herbal medicine.

## Data availability statement

The microbiomics data presented in the study are deposited in the Sequence Read Archive repository (https://www.ncbi.nlm.nih.gov/), accession number PRJNA904900. The metabolomics data presented in the study are deposited in the MetaboLights Study repository (https://www.ebi.ac.uk/metabolights/), accession number MTBLS6642. The protein data are deposited at (https://www.iprox.cn/page/PSV023.html;?url=1671194978436ARUz) password: NGt5.

## Ethics statement

The animal study was reviewed and approved by Ethics Committee of Affiliated Hospital of Qingdao University.

## Author contributions

DL: formal analysis, software, and manuscript writing – original draft. H-JY: investigation and data curation. G-JH: formal analysis, data curation, and methodology. R-YY: investigation. A-MX: supervision. X-YL: conceptualization, funding acquisition, project administration, and writing review and final approval of manuscript. All authors contributed to the article and approved the submitted version.

## Footnotes

1
www.theplantlist.org

2
http://herb.ac.cn/

3
https://www.kegg.jp/kegg/
