# Supplementary material for: Mechanisms of the Ping-wei-san plus herbal decoction against Parkinson’s disease: Multiomics analyses
Source: Front Nutr. 2023 Jan 4;9:945356. doi: 10.3389/fnut.2022.945356 (PMC9845696; doi:10.3389/fnut.2022.945356)

Each WB contains 9 bands, and the 3rd to 9th bands in each image are used for this publication. Bands 1 and 2 of each picture are another set of fasting treatments that have not been included in the content of this article.

For example, the following picture is the WB picture of expression of TH in substantia nigra.

For each picture:

3-4 the control group;

5-6 the PD group;

7-9 the PWP group and the L-Dopa group.


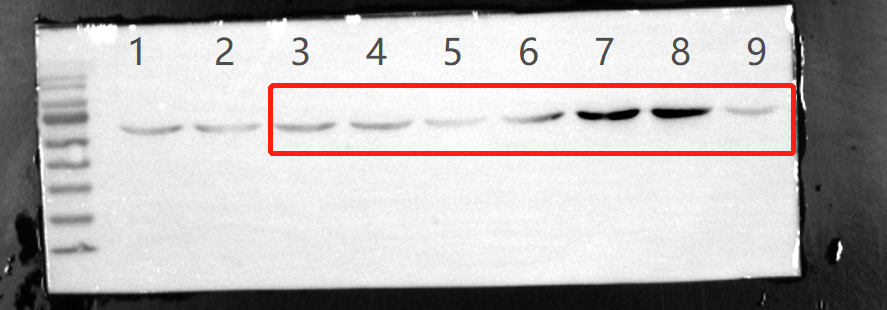


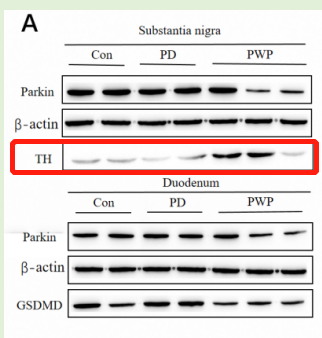

Supplement: Supplementary file 1 [file Data_Sheet_1.docx]
