# Supplementary figures and images for: Mechanisms of the Ping-wei-san plus herbal decoction against Parkinson’s disease: Multiomics analyses
Source: Front Nutr. 2023 Jan 4;9:945356. doi: 10.3389/fnut.2022.945356 (PMC9845696; doi:10.3389/fnut.2022.945356)

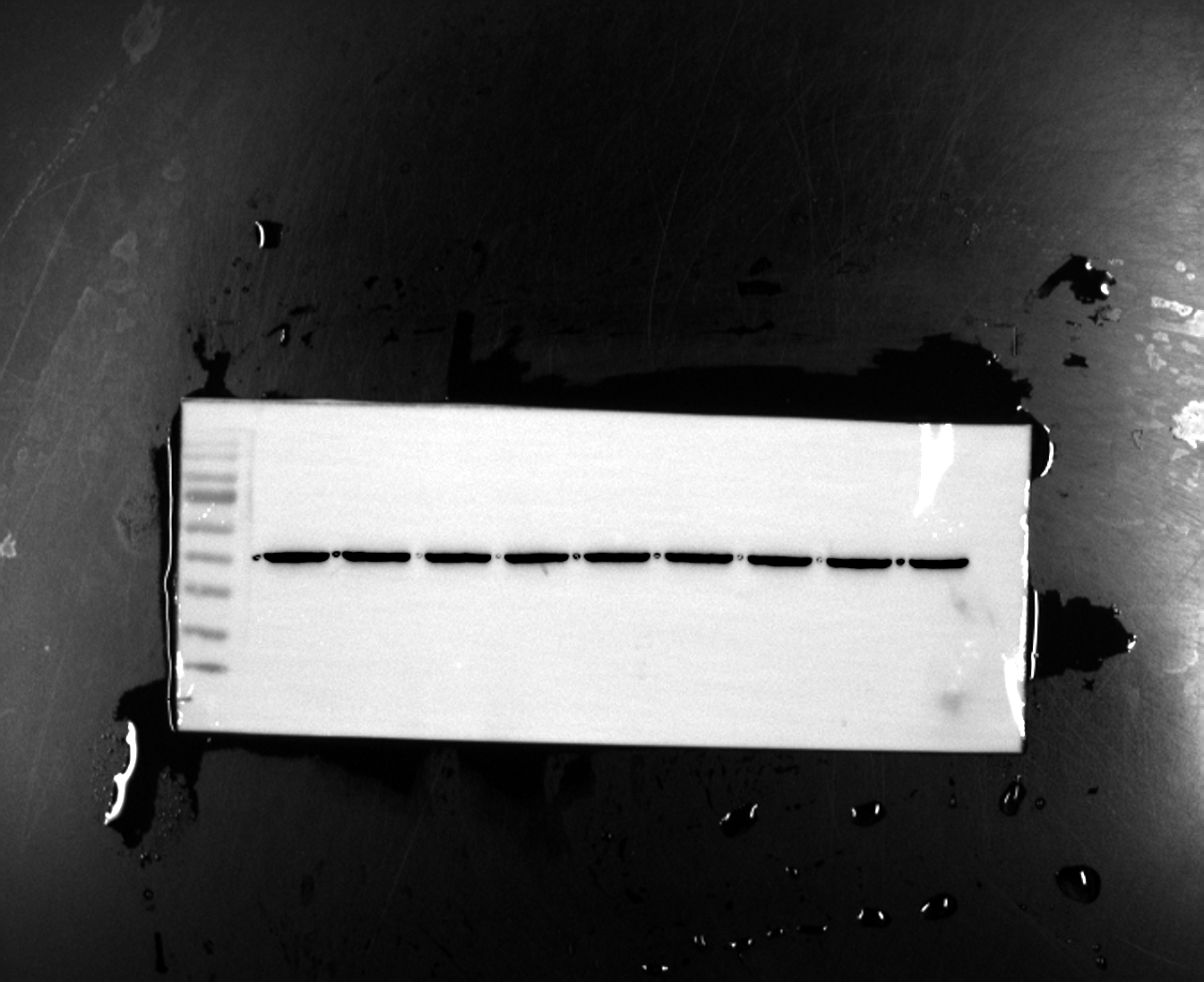

Supplement: Supplementary file 2 [file Image_1.TIF]

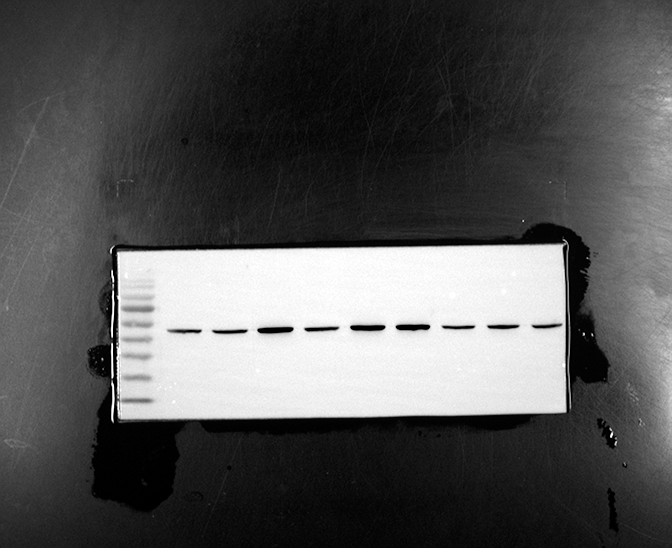

Supplement: Supplementary file 3 [file Image_2.TIF]

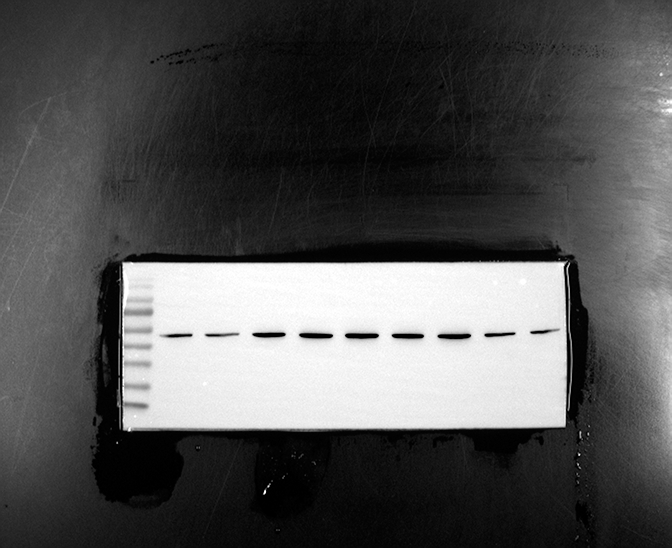

Supplement: Supplementary file 4 [file Image_3.TIF]

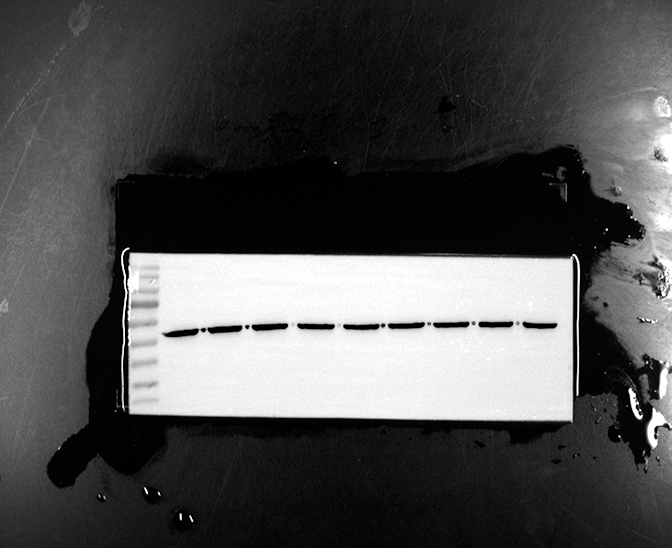

Supplement: Supplementary file 5 [file Image_4.TIF]

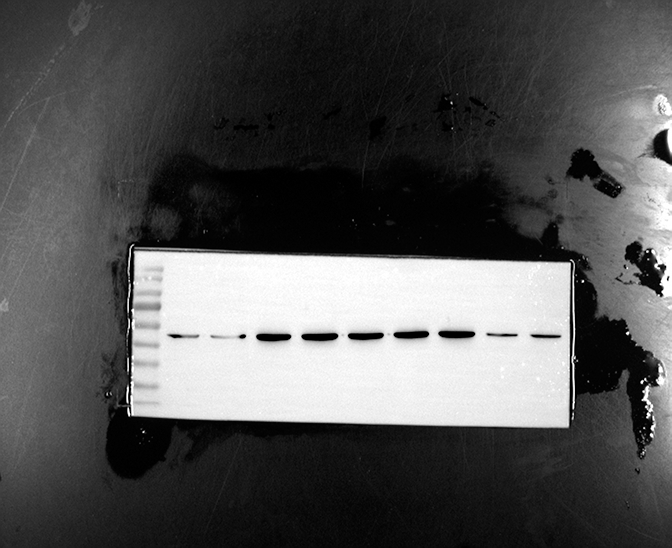

Supplement: Supplementary file 6 [file Image_5.TIF]

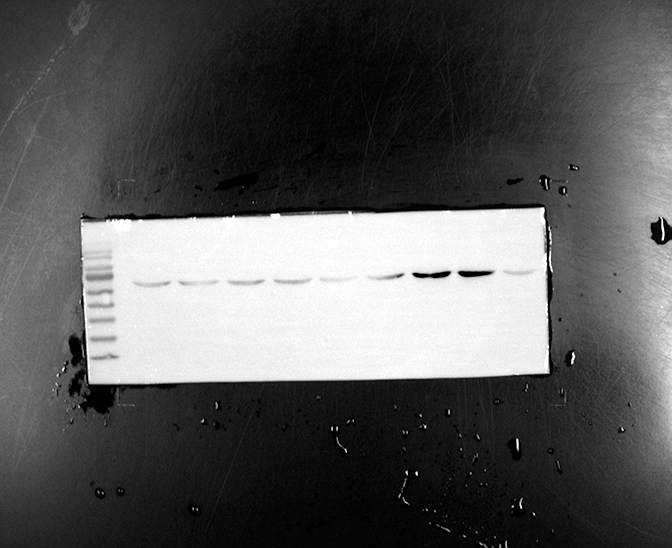

Supplement: Supplementary file 7 [file Image_6.TIF]
